# Functional Cooperation between KCa3.1 and TRPV4 Channels in Bronchial Smooth Muscle Cell Proliferation Associated with Chronic Asthma

**DOI:** 10.3389/fphar.2017.00559

**Published:** 2017-08-25

**Authors:** Zhihua Yu, Yanxia Wang, Lu Qin, Hongzhuan Chen

**Affiliations:** Department of Pharmacology and Chemical Biology, Shanghai Jiao Tong University School of Medicine Shanghai, China

**Keywords:** calcium, bronchial smooth muscle, airway remodeling, mouse, culture

## Abstract

Airway smooth muscle cells (SMC) proliferation contributes to the airways remodeling and irreversible airway obstruction during severe asthma, but the mechanisms of airway SMC proliferation are poorly understood. Intracellular Ca^2^**^+^** levels play an important role in regulating cell proliferation. We have previously reported KCa3.1 channels regulated human bronchial smooth muscle (HBSM) cells proliferation via the Ca^2+^ influx as a consequence of membrane hyperpolarization. However, the role of potassium channels KCa3.1 in airway remodeling as well as the mechanism for extracellular Ca^2^**^+^** influx induced by the activation of KCa3.1 remains unknown. Here we demonstrated that KCa3.1 channels deficiency attenuated airway remodeling, airway inflammation, and airway hyperresponsiveness (AHR) in a mouse model of chronic asthma. The gene expressions of repressor element 1-silencing transcription factor (REST) and c-Jun, two transcriptional regulators of KCa3.1 channels, were correlated negatively or positively with KCa3.1 channels expressions both *in vivo* and *in vitro* using real-time PCR and Western blot analyses. RNAi-mediated knockdown or pharmacological blockade of KCa3.1 and TRPV4 significantly attenuated HBSM cells proliferation. Using confocal imaging and custom data analysis software, blockade of TRPV4 decreased the Ca^2^**^+^** influx induced by 1-EBIO-mediated KCa3.1 activation. Double-labeled staining showed that KCa3.1 and TRPV4 channels colocalized in HBSM cells. These results demonstrate that KCa3.1 channels regulate the proliferation phenotype of HBSM cells via TRPV4 channels in the process of chronic asthma, making it a potential therapeutic target to treat chronic asthma.

## Introduction

Chronic asthma is an inflammatory airway disorder characterized by AHR and airway remodeling (Lim et al., 2017). Increased mucous gland hyperplasia, extracellular matrix deposition, and the proliferation of airway SMC may result in lung function decline (Wagenaar et al., 2015). Increased bulk of HBSM plays an important role in airway remodeling derived from subjects with asthma. Asthmatic airway inflammatory mediators (e.g., platelet-derived growth factor, PDGF) are HBSM cells mitogens, and increased proliferation and migration of HBSM cells in response to mitogens is a crucial event in airway structural remodeling (Zou et al., 2014).

The intermediate conductance calcium-activated potassium channel KCa3.1 is highly expressed in a variety of non-excitable cells, such as T lymphocytes, B cells, mast cells, HBSM cells and fibroblasts (Wei et al., 2016; Staal et al., 2017). A small rise in free cytosolic Ca^2+^ ([Ca^2+^]*_i_*) can activate the channel, which can induce membrane hyperpolarization and Ca^2+^ influx required for cell activation, proliferation and migration. Previously, we reported that blockade of KCa3.1 inhibited OVA-induced airway inflammation, airway remodeling and AHR in a mouse model of chronic asthma (Yu et al., 2013b). 1-((2-chlorophenyl) (diphenyl) methyl)-1H-pyrazole (TRAM-34) or KCa3.1 gene silencing suppressed the asthmatic HBSM cell proliferation and migration through influences on membrane potential and Ca^2+^ signaling (Yu et al., 2013a). In proliferative phenotype of HBSM cells, up-regulation of KCa3.1 promoted HBSM cells phenotypic modulation in response to PDGF stimuli by regulating membrane hyperpolarization.

The KCa3.1 gene promoter contains the transcription factors AP-1 binding site, which is associated with KCa3.1 induction during T cells activation (Bertuccio et al., 2014; Ferreira et al., 2014). Repressor element-1 silencing transcription factor (REST) was reported to suppress KCa3.1 in contractile phenotype vascular SMC (Tharp et al., 2008; Choi et al., 2013; Ohya et al., 2016). AP-1 and REST may coordinate for KCa3.1 induction in HBSM cells. Although Ca^2+^ influx involved in the proliferation of HBSM cells via intracellular signaling pathways, the molecular mechanism by which KCa3.1 regulates HBSM cells proliferation remains unknown. KCa3.1 genetic deficiency protected against pulmonary circulatory collapse and lung damage induced by activation of calcium-permeable transient receptor potential vanilloid 4 (TRPV4) channel (Simonsen et al., 2017). TRPV4 and KCa3.1 also functionally couple as osmosensors in the paraventricular nucleus (Feetham et al., 2015).

In the present study, we demonstrated that KCa3.1 deficiency resulted in the attenuation of airway remodeling, inflammation, and AHR in a mouse model of chronic asthma. KCa3.1 knockdown suppressed HBSM cells proliferation by inhibiting the [Ca^2+^]_i_ influx via TRPV4 channels and subsequent mitogenic signaling pathways *in vitro.* For the first time, we show an endogenous interaction between KCa3.1 and TRPV4 in HBSM cells. These results strongly suggest that KCa3.1 channels play an important role in the process of HBSM cells proliferation via TRPV4 channels in chronic asthma, making it a potential therapeutic target to treating chronic asthma.

## Materials and Methods

### Mice

All animal experiments were approved by the Animal Experimentation Ethics Committee of Shanghai Jiao Tong University School of Medicine (ethics protocol number: A-2015-010). KCa3.1 gene deletion (KCa3.1^-/-^, KO) mice were obtained from the Jackson Laboratory. 8–10 week old wild type (KCa3.1^+/+^, WT) and KO male mice were housed in a specific pathogen-free animal facility with free access to food and water.

### Allergen-Induced Airway Inflammation and Remodeling

The chronic asthmatic mice model was described previously (Yu et al., 2013b). Briefly, on Days 1 and 14, WT and KO mice were sensitized by an intraperitoneal (i.p.) injection of 20 mg ovalbumin (OVA, Grade V; Sigma–Aldrich) emulsified in 2.25 mg of aluminum hydroxide in phosphate buffered saline (PBS). Then the WT and KO mice were challenged with aerosolized 5% OVA on Days 21, three times per week for the following 8 weeks. These WT + OVA group mice (OVA-sensitized/challenged WT mice) and KO + OVA group mice (OVA-sensitized/challenged KO mice) were used as the model of chronic asthma. The control groups of WT and KO mice were sensitized with PBS plus aluminum hydroxide and challenged with PBS. After the final OVA challenge, airway isometric tension was assessed, and the lung samples were obtained for further analysis.

### Measurement of Airway Isometric Tension

The mice were anesthetized with chloral hydrate and then the bronchus was removed as described previously (Xu et al., 2011). Briefly, bronchial rings were mounted in a modified Krebs solution (composition in mM: glucose 11, CaCl_2_ 2.5; NaCl 118; KCl 4.7; NaHCO_3_ 25; MgSO_4_ 1.2; KH_2_PO_4_ 1.2; EDTA⋅Na_2_ 0.5), maintained bubbled with a mixed gas of 95% O_2_ and 5% CO_2_ at 37°C. PowerLab 8sp Life Analysis System was used to record isometric tension (Ad Instruments Australia). A resting tension of 500 mg was used as the preparations connected vertically to a force-displacement transducer for 1 h. Preparations were washed every 15 min for 1 h until the cumulative concentration-response curves of methacholine were measured. The changes of the four groups (WT, WT + OVA, KO, KO + OVA) airway isometric tension were expressed as isometric force (mg) and EC_50_, i.e., the concentration of methacholine causing 50% of the maximal force generated (EC_50_). The EC_50_ was calculated from the logarithmic regression of each concentration–response curve.

### Lung Tissue Histopathology

Airway inflammatory cell counts were scored on a five-point scale as described previously: 0 = no cells, 1 = a few cells, 2 = a ring of cells one cell layer deep, 3 = a ring of cells two to four cells deep, and 4 = a ring of cells of more than four cells deep (Yu et al., 2013b). Periodic acid-Schiff (PAS)-stained goblet cells of each bronchus epithelium were scored based on a 5-point scoring system: 0, <5% goblet cells, 1, 5–25%, 2, 25–50%, 3, 50–75%, and 4, >75% (Yu et al., 2013b). At least three different fields of each lung section were performed to score the inflammatory cells and mucus production. Mean scores were obtained from five animals. Image J software (Schneider et al., 2012) was used to quantify Masson’s trichrome staining intensity.

### Immunostaining and Data Analysis

For immunofluorescence staining of lung sections and cultured asthmatic HBSM cells, the tissues and cells were blocked with 3% bovine serum albumin in PBS 1 h at room temperature. Sections and cells were incubated at 4°C overnight with primary antibodies: mouse anti-KCa3.1 (1:100; Alomone labs), rabbit anti-TRPV4 (1:200; Alomone labs), mouse anti-α-SMA (1:400, Sigma, MO, United States). The sections and cells were then incubated with following secondary antibodies: Alexa Fluor 555 goat anti-rabbit IgG and Alexa Fluor 488 goat anti-mouse IgG (1:500; Invitrogen) for 1 h at room temperature. Then washed with PBS and mounted with DAPI. Immunofluorescence was visualized by a Leica TCS SP8 Confocal Laser Scanning Microscope (Leica, Germany).

Quantitative evaluation of sections stained with α-SMA antibody was performed using Leica LAS AF Lite software (Leica, Germany). All analyses were done blinded to the treatment groups. Results were shown as area of α-SMA labeling per micrometer length of bronchi’s basement membrane in internal diameter as described previously (Qin et al., 2012). At least 6 bronchi were counted on each slice.

Analysis of colocalization: For analysis of TRPV4 and KCa3.1 colocalization, images were processed and analyzed using Leica LAS AF Lite software (Leica, Germany). Pearson’s correlation was used to express the degree of colocalization as described early (Wu et al., 2012; Bradley et al., 2015). The colocalization of KCa3.1 and TRPV4 in the overlap of the two channels was assessed using the colocalization tool in Leica LAS AF Lite software. The Pearson correlation values range from -1 to +1. A correlation of 1 indicates complete colocalization between the two proteins. A correlation of -1 indicates a negative interaction, and a correlation of 0 indicates no colocalization between the two proteins (Wu et al., 2012).

### Real-Time PCR

Lung tissues of different groups mice were frozen in TRIzolsolution (Invitrogen, Carlsbad, CA, United States). According to the protocol of TRIzol, total RNA was isolated and transcribed to cDNA using the RevertAid^TM^ First Strand cDNA Synthesis Kit (Fermentas, Glen Burnie, MD, United States). The primer sequences were as follows: for REST,

5′-TTCAAATCTAAGCATCCCACCT-3′ (sense) and
5′-TTCAAATCTAAGCATCCCACCT-3′ (antisense); for c-Fos,
5′-TTCAAATCTAAGCATCCCACCT-3′ (sense) and
5′-TCAGGGTAGGTGAAGACAAAGG-3′ (antisense); for FosB,
5′-GTCAACATCCGCTAAGGAAGAC-3′ (sense) and
5′-GTCAACATCCGCTAAGGAAGAC-3′ (antisense); for Fra2,
5′-GTCAACATCCGCTAAGGAAGAC-3′ (sense) and
5′-TGAGCCACCAACATGAACTCTA-3′ (antisense); for Fra1,
5′-CAAAATCCCAGAAGGAGACAAG-3′ (sense) and
5′-AAAAGGAGTCAGAGAGGGTGTG-3′ (antisense); for c-Jun,
5′-TATGACTGCAAAGATGGAACG-3′ (sense) and
5′-AGGTTCAAGGTCATGCTCTGTT-3′ (antisense); for JunB,
5′-GCCTTTCTATCACGACGACTCT-3′ (sense) and
5′-GGGTTTCAGGAGTTTGTAGTCG-3′ (antisense); for JunD,
5′-ACCAGTACGCAGTTCCTCTACC-3′ (sense) and
5′-CTTTGCTTGTGCAGGTCCTC-3′ (antisense); for β-actin,
5′-CATCCGTAAAGACCTCTATGCCAAC-3′ (sense) and
5′-ATGGAGCCACCGATCCACA-3′ (antisense).

SYBR Green I was used to perform quantitative real-time PCR on an ABI 7500 sequence detector system (Applied Biosystems). Target gene expression was normalized to β-actin using the 2^-ΔΔCT^ method.

### Enzyme-Linked Immunosorbent Assay

Bronchoalveolar lavage fluid (1 mM EDTA, 10% FBS, 0.01M PBS) was used to lavage the lung tissues through the tracheal tube. ELISA was performed using the kit for interferon-γ (IFN-γ), interleukin (IL)-4, IL-5, and IL-13 (Rapidbio Labs, Langka Trade Co., Ltd., Shanghai, China).

### Asthmatic HBSM Cells Culture

Asthmatic HBSM cells (Lonza, Walkersville, MD, United States) were described previously (Yu et al., 2013b). The cells between Passages 4 and 8 were used. HBSM cells were cultured in Dulbecco’s Modified Eagle’s medium (DMEM) media containing 10% fetal bovine serum (FBS) and 100 U/ml penicillin/streptomycin at 1 × 10^5^ cells/cm^2^. HBSM cells were serum-free for 24 h for a quiescent state, and were then treated with 20 ng/mL PDGF (ProSpec, ProSpec-Tany TechnoGene Ltd., Israel). In some cases, HBSM cells were pretreated with TRAM-34 or HC 067047 (Tocris Bioscience, United Kingdom) 1 h before PDGF was added.

### Transfection of siRNA

The transfection of small interfering RNA (siRNA) was performed as described previously (Yu et al., 2013b). Briefly, HBSM cells were 70–80% confluent 24 h before transfection. HBSM cells were transfected with KCa3.1- and TRPV4-specific siRNA and with scrambled siRNA as a negative control (siCTL). TurboFect siRNA Transfection Reagent (Fermentas) was used to incubate the cells for 72 h after transfection.

### Intracellular Free Calcium Measurement

Leica TCS SP8 Confocal Laser Scanning Microscope (Leica, Germany) was used to evaluate the relative changes in [Ca^2+^]_i_ by monitoring Fluo-4 fluorescence. 3 μM Fluo-4 AM (Sigma–Aldrich) was used to stain the asthmatic HBSM cells for 30 min at 37°C before measuring [Ca^2+^]_i_. 200 μM 1-EBIO was added to the cells. Fluo-4 fluorescence was measured at 510 nm, with excitation at 488 nm. Confocal images were taken every 2 s for 360 s. Representative curves were used to show the fold changes in [Ca^2+^]_i_ relative to the unstimulated condition over 360 s.

### Western Blotting

The lysates of tissues or cells were homogenized in RIPA buffer (25 mM Tris pH 7.4, 150 mM NaCl, 1% NP-40, 0.1% sodium dodecyl sulfate, 4% protease inhibitor). The following primary antibodies were used: anti-TRPV4 (1:500; Alomone Labs), anti-KCa3.1 (1:500; Abcam), anti-α-SMA (1:1000; Sigma), anti-phospho-JNK/c-Jun antibodies (1:1000, Cell Signaling Technology, Danvers, MA, United States), anti-REST (1:500; Santa Cruz, Santa Cruz, CA, United States), and anti-β-actin (1:1000; Santa Cruz). HRP-conjugated anti-rabbit or anti-mouse IgG secondary antibodies (1:3000; Amersham Biosciences) were used for 1 h at room temperature. Bands were quantified by ImageJ software and normalized to the β-actin band as loading control (Schneider et al., 2012).

### Cell Proliferation

Cell proliferation assay was performed as reported previously (Cell Counting Kit-8, CCK-8, Dojindo Laboratories, Kumamoto, Japan) (Yu et al., 2013b). HBSM cells were serum-free for 24 h, and then the HBSM cells were pretreated with the blockers for 1 h before 10% FBS was added. All control experiments were performed in the presence of vehicle for the blockers.

### Statistical Analysis

All data are presented as means ± SEM. Statistical analyses were performed using Prism software (GraphPad Software, Inc., La Jolla, CA, United States). Data were tested for Gaussian distribution with the Kolmogorov–Smirnov normality test and then analyzed by one-way ANOVA and Dunnett’s *post hoc* tests. For AHR comparisons, two-way ANOVA with a Bonferroni post test was used. Non-parametric functional outcome scores were compared by the Kruskal–Wallis test with Dunn’s *post hoc* tests. Data were analyzed with unpaired, two-tailed Student’s *t*-test when comparing between two groups, or the non-parametric Mann–Whitney test was applied. Statistical significance was set at *p* < 0.05.

## Results

### Gene Deletion of KCa3.1 Attenuates AHR

**FIGURE 1:**
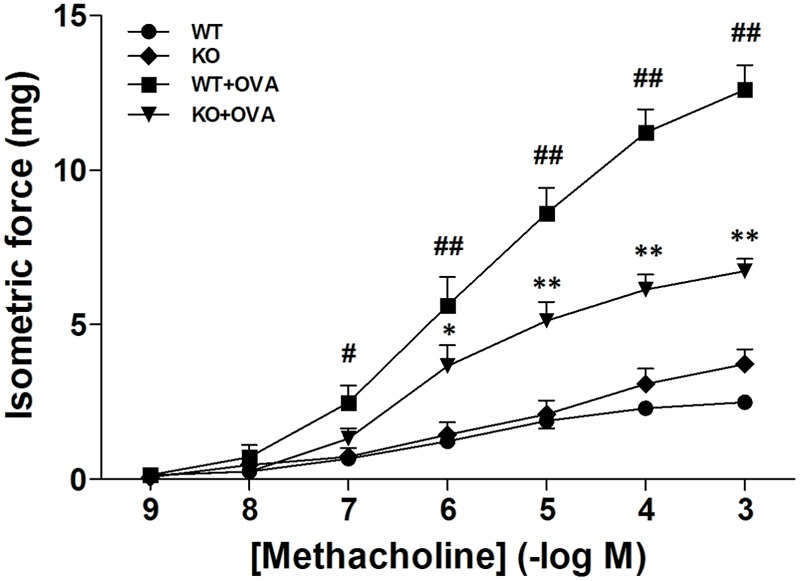
Gene deletion of KCa3.1 reduced established OVA-induced changes in constrictor responsiveness to methacholine in mouse bronchi. Relative to WT group bronchi, maximal isometric contractile force responses to methacholine were significantly increased in WT + OVA group bronchi, and this increased constrictor responsiveness to methacholine was prevented in KO + OVA group bronchi tissues. Data represented means ± SEM (*n* = 4–6). ^#^*p* < 0.05, ^##^*p* < 0.01 versus WT mice. ^∗^*p* < 0.05, ^∗∗^*p* < 0.01, two-way ANOVA followed by the Bonferroni multiple comparison test compared with WT + OVA group mice. WT, wildtype; KO, knockout.

We previously reported that pharmacological blockade of KCa3.1 dose-dependently attenuated the airway AHR in a mouse model of chronic asthma (Yu et al., 2013b). Here we tested whether gene deletion of KCa3.1 exerted beneficial effect on airway AHR in the mouse model of chronic asthma. **Figure 1** showed the responses of the WT, KO, WT + OVA, and KO + OVA groups’ bronchi to methacholine accumulating concentration. The methacholine concentration-response curve of bronchial preparations from the WT + OVA group mice was markedly shifted upward compared with that from the WT group mice (**Figure 1**, *p* < 0.01). Sensitivity to methacholine, however, was not affected (EC_50_ 5.9 ± 0.3 mg and 5.7 ± 0.3 mg for WT group and WT + OVA group mice, respectively). In contrast, KO + OVA group significantly attenuated the up-regulation of contractility compared to the WT + OVA group (**Figure 1**, *p* < 0.01). The sensitivity to methacholine, was also not altered (KO + OVA group EC_50_ 6.0 ± 0.4 mg).

### Gene Deletion of KCa3.1 Attenuated Established Airway Inflammation

**FIGURE 2:**
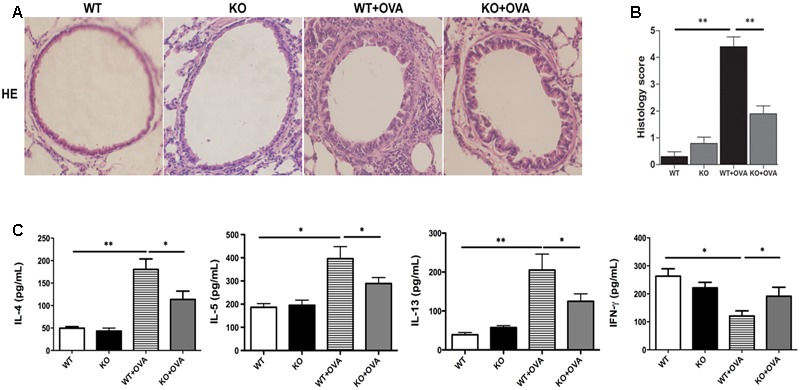
Gene deletion of KCa3.1 reduced established airway inflammation. **(A)** Representative images of hematoxylin and eosin-stained lung sections from WT, KO, WT + OVA, and KO + OVA groups (original magnification, X30). **(B)** Quantitative analyses of inflammatory cell infiltration in lung sections. Data were expressed as means ± SEM (*n* = 5). ^∗∗^*p* < 0.01, one-way ANOVA followed by the Dunnett’s multiple comparison test compared with WT + OVA mice. **(C)** Effects of KCa3.1 deficiency on BAL fluid cytokine concentrations in a mouse model of chronic asthma. BAL fluid cytokine concentrations from WT, KO, WT + OVA, and KO + OVA groups. Concentrations of IL-4, IL-5, IL-13, and IFN-γ were analyzed using ELISA. Values were shown as means ± SEM (*n* = 6). ^∗^*p* < 0.05, ^∗∗^*p* < 0.01, one-way ANOVA followed by the Dunnett’s multiple comparison test compared with WT + OVA mice. WT, wildtype; KO, knockout.

Inflammatory cells recruitment in the airway is one of the characteristic features in the development of asthmatic pathophysiology. As shown in **Figure 2**, gene deletion of KCa3.1 exerted beneficial effects on airway inflammation in the mouse model of chronic asthma. Hematoxylin and eosin-stained lung tissues showed that gene deletion of KCa3.1 significantly reduced the inflammatory influx into the lung tissue in the KO + OVA group mice compared to the WT + OVA group mice (**Figures 2A,B**, *p* < 0.01).

It was well known that the aberrant production of the T helper cell type 2 (Th2) cytokines played an important role in allergic asthma. Th2 cells cytokines participated in airway inflammation via B cell activation, eosinophil proliferation, and secretion of inflammatory components. Th1 cytokines such as IFN-γ were well known to inhibit the production of Th2 cytokines, which participated in the process of asthma. The imbalance of Th1 and Th2 cytokines has been reported to be involved in the allergic asthma (Bui et al., 2017). Thus Th2 and Th1-type cytokines concentrations were measured in bronchoalveolar lavage fluid to investigate whether KCa3.1 was involved in OVA-induced dominant T cell-derived cytokines. The concentrations of Th2-type cytokines (IL-4, IL-5, and IL-13) appeared to be lower in WT group mice. As we expected, WT + OVA group mice showed up-regulation of Th2-type cytokines IL-4, IL-5, and IL-13 compared to WT group (**Figure 2C**). In contrast, WT + OVA group mice showed decreased concentration of the Th1-type cytokine IFN-γ compared to WT group (**Figure 2C**). Interestingly, gene deletion of KCa3.1 (KO + OVA group) significantly attenuated the up-regulation of IL-4, IL-5, and IL-13 and increased concentrations of IFN-γ, in comparison with WT + OVA group mice (**Figure 2C**, *p* < 0.01).

### Gene Deletion of KCa3.1 Prevented Airway Remodeling

**FIGURE 3:**
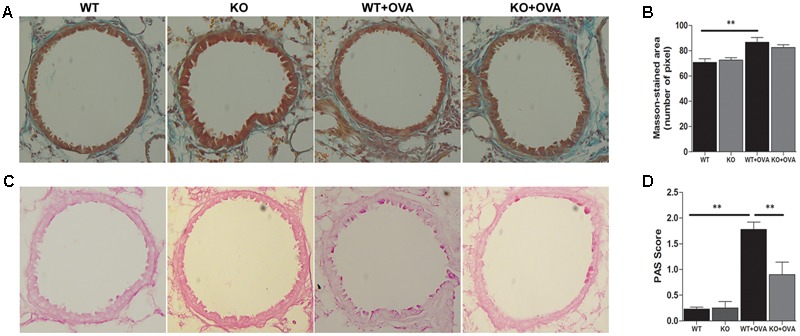
Gene deletion of KCa3.1 prevented established airway remodeling. **(A)** Representative images were obtained of Masson trichrome-stained lung sections from WT, KO, WT + OVA, and KO + OVA groups (original magnification, X 30). **(B)** Random measurements from the basement membrane into the submucosa (10 measurements of 20 μm in length) were taken, and the mean density was calculated from four bronchi per mouse (*n* = 5). ^∗∗^*p* < 0.01, one-way ANOVA followed by the Dunnett’s multiple comparison test compared with WT + OVA mice. **(C)** Representative images were obtained of PAS-stained lung sections from WT, KO, WT + OVA, and KO + OVA groups (original magnification, X 30). **(D)** PAS-stained sections were scored. Data were presented as means ± SEM (*n* = 5). ^∗∗^*p* < 0.01, one-way ANOVA followed by the Dunnett’s multiple comparison test compared with WT + OVA mice. WT, wildtype; KO, knockout.

To study whether gene deletion of KCa3.1 channels can attenuate airway remodeling in the mouse model of chronic asthma, as measured by Masson trichrome staining, PAS staining and α-SMA expression, in comparison with WT + OVA mice. Firstly, images analyses were performed to measure the matrix density of lung sections from WT, KO, WT + OVA, and KO + OVA groups of mice. Matrix deposition was significantly increased in the WT + OVA group mice compared with WT group mice (**Figures 3A,B**, *p* < 0.01). While there was no significantly decreased of matrix deposition in the KO + OVA group mice compared with WT + OVA group mice (**Figures 3A,B**, *p* > 0.05). PAS staining was used to quantify mucus-secreting goblet. Gene deletion of KCa3.1 significantly decreased the number of mucus-secreting goblet cells in the KO + OVA group mice compared to the WT + OVA group mice (**Figures 3C,D**, *p* < 0.01).

**FIGURE 4:**
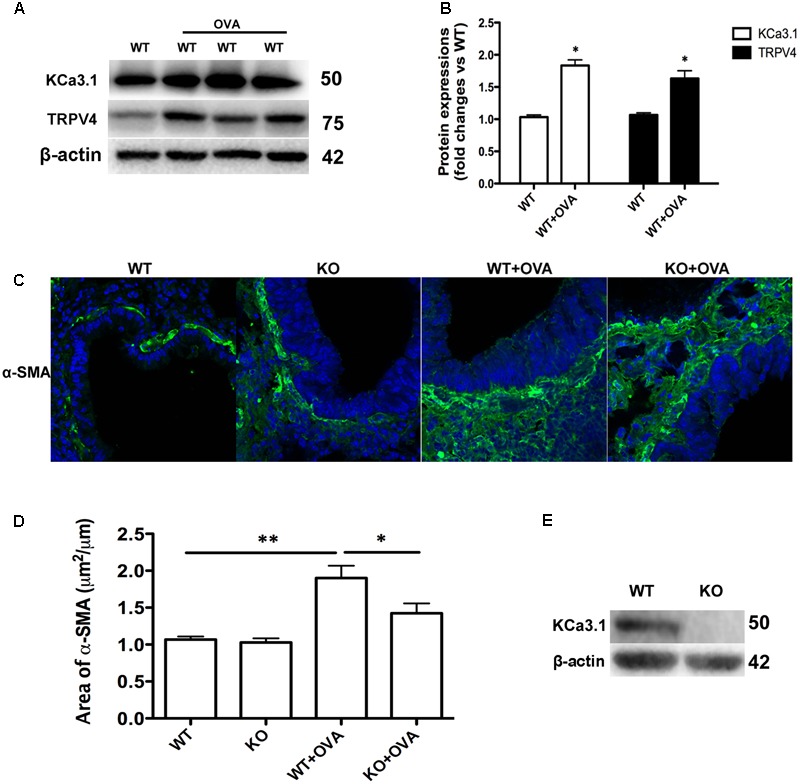
Gene deletion of KCa3.1 prevented OVA-induced up-regulation of α-SMA. **(A)** The KCa3.1 and TRPV4 proteins expressions in the lung sections from WT and WT + OVA groups mice. **(B)** Data were presented as means ± SEM (*n* = 5). ^∗^*p* < 0.05, unpaired, two-tailed Student’s *t*-test compared with WT mice. **(C)** Representative photomicrographs of immunofluorescence reactivity of lung sections from WT, KO, WT + OVA, and KO + OVA groups mice using antibody against α-SMA (original magnification, x 40). **(D)** Plot of means ± SEM measurements of the area α-SMA^+^ staining per μm length of bronchi basement membrane. *n* = 4, ^∗^*p* < 0.05, ^∗∗^*p* < 0.01, Kruskal–Wallis test followed by Dunn’s multiple comparison test compared with WT + OVA mice. **(E)** Western blot showing the absence of KCa3.1 expression in the lung tissues of KCa3.1 KO mice. WT, wildtype; KO, knockout.

The expressions of KCa3.1 and TRPV4 proteins were measured in WT and WT + OVA group mice bronchi, using Western blot analyses. The expressions of KCa3.1 and TRPV4 channels in WT + OVA group mice were approximately 2-fold and 1.5-fold higher than those in WT group mice, respectively (**Figures 4A,B**, *p* < 0.05).

Enhanced smooth muscle mass is also a pathogenic feature of asthmatic airway remodeling. We detected α-SMA expression of lung sections from WT, KO, WT + OVA, and KO + OVA group mice. As shown in **Figure 4, OVA sensitization and challenge** induced up-regulation of α-SMA protein staining in the airways of WT + OVA group mice compared to the WT group mice (**Figures 4C,D**, *p* < 0.01). KO + OVA group mice exhibited significantly less α-SMA staining (per μm of bronchi basement membrane) relative to WT + OVA group mice (**Figures 4C,D**, *p* < 0.05). However, the levels of airway α-SMA protein staining in the non-OVA challenged mice were similar in both the WT and KO group mice (**Figures 4C,D**). The absence of KCa3.1 in lung tissues was confirmed by examining KCa3.1 protein expression using Western blotting (**Figure 4E**). These data suggested that KCa3.1 and TRPV4 channels involved in airway remodeling in the process of chronic asthma.

### Expressions of KCa3.1 Transcriptional Regulators in Chronic Asthma

**FIGURE 5:**
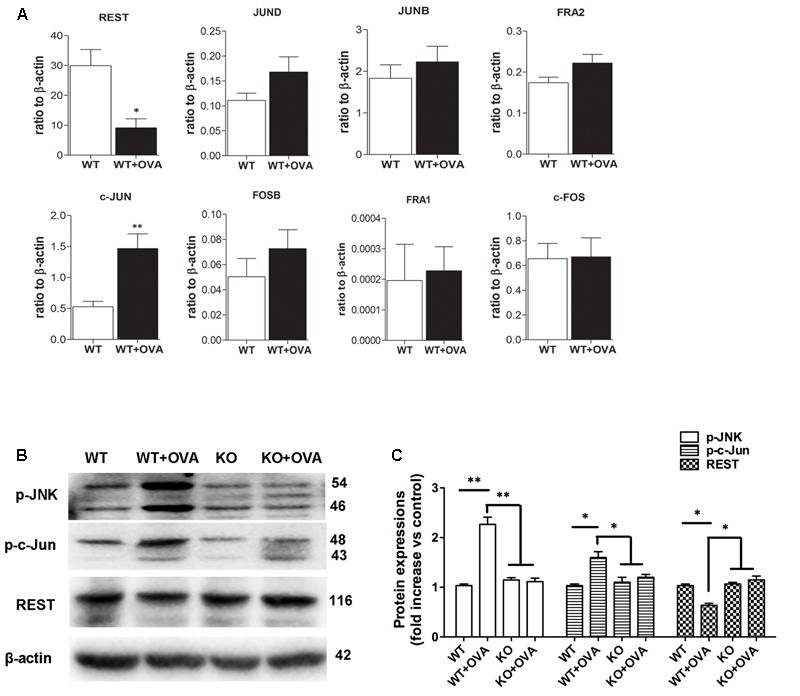
Gene and protein expressions of REST and AP-1 components. **(A)** Real-time PCR analysis of REST, JunD, JunB, Fra2, c-Jun, FosB, Fra1, and c-Fos in WT and WT + OVA group mice. Values were shown for steady-state transcripts relative to β-actin. Data were presented as means ± SEM.^∗^*p* < 0.05, ^∗∗^*p* < 0.01 versus WT group (*n* = 5), Mann-Whitney test compared with WT mice. **(B)** p-JNK, p-c-Jun, and REST protein expressions in WT, WT + OVA, KO, and KO + OVA group mice. **(C)** Data were presented as means ± SEM. *n* = 5, ^∗^*p* < 0.05, ^∗∗^*p* < 0.01, one-way ANOVA followed by the Dunnett’s multiple comparison test compared with WT + OVA mice. WT, wildtype; KO, knockout.

It was previously reported that up-regulation of KCa3.1, whose expression was suppressed by REST and also regulated by nuclear transcription factor AP-1 (Fos/Jun) (Wisdom, 1999; Ghanshani et al., 2000; Cheong et al., 2005), was necessary for porcine coronary SMC migration *in vitro* (Tharp et al., 2006). The expressions of AP-1 (Fos/Jun) members and REST were determined, using real-time PCR analysis. As shown in **Figure 5A**, the expression of REST transcript was significantly lower found in WT + OVA group mice than that in the WT group bronchi (**Figure 5A**, *p* < 0.05). However, the expression of c-Jun transcript was significantly higher in WT + OVA group than that in the WT group bronchi (**Figure 5A**, *p* < 0.01). No significant differences were detected in the expressions of c-Fos, FosB, Fra-1, Fra-2, JunB, and JunD between these two groups (**Figure 5A**, *p* > 0.05).

c-Jun N-terminal kinases (JNK) were shown to bind and phosphorylate c-Jun within its transcriptional activation domain. JNK pathway was activated by different stressing factors, and played an important role in cell death, gene expression, and regulation of cellular senescence (Yarza et al., 2016). The expressions of JNK/c-Jun and REST proteins in the bronchi of WT, KO, WT + OVA, and KO + OVA group mice were determined by Western blot analysis. Anti-JNK, anti-c-Jun and anti-REST antibodies were used to detect JNK, c-Jun and REST proteins from the WT, KO, WT + OVA, and KO + OVA four groups mice. Western blot analysis revealed that JNK/c-Jun and REST expression levels were significantly higher and lower in WT+OVA group than those in WT group (**Figures 5B,C**). In the group of KO + OVA mice, gene deletion of KCa3.1 decreased the expression of JNK/c-Jun and increased the expression of REST expression compared to the WT + OVA group (**Figures 5B,C**).

### Activation of KCa3.1 Induces Ca^2+^ Influx through TRPV4 Channels

It was demonstrated genetic deficiency of KCa3.1 protected against lung damage induced by activation of TRPV4 channel (Simonsen et al., 2017). TRPV4 and KCa3.1 also functionally couple as osmosensors in the paraventricular nucleus (Feetham et al., 2015). Our research had shown that KCa3.1 regulated Ca^2+^ influx via membrane hyperpolarization in the HBSM cells (Yu et al., 2013b).

**FIGURE 6:**
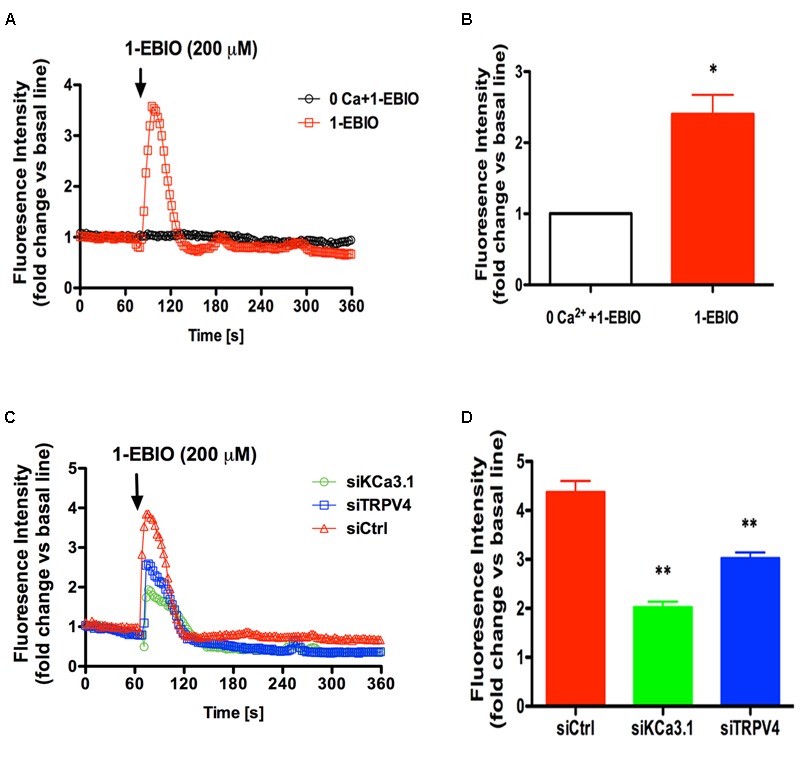
TRPV4 involved in Ca**^2+^** entry induced by KCa3.1 activation in asthmatic HBSM cells. **(A,C)** Representative curves showed the fold changes in [Ca^2+^]_i_ relative to the unstimulated condition over 360 s. **(B,D)** The bar graphs indicated the maximal increase in [Ca^2+^]_i_ mobilization. **(A)** External calcium was required for 200 μM 1-EBIO-induced Ca**^2+^** elevations. The increase of [Ca**^2+^**]_i_ induced by 200 μM 1-EBIO was prevented by 0 external Ca**^2+^**. **(B)** Quantitative analysis of 1-EBIO-induced Ca**^2+^** entry. *n* = 10–20, ^∗^*p* < 0.05, unpaired, two-tailed Student’s *t*-test compared with 0 Ca**^2+^ +** 1-EBIO. **(C)** Representative traces of 1-EBIO-induced Ca**^2+^** entry in. 1-EBIO was used at 200 μM. **(D)** Quantitative analysis of 1-EBIO-induced Ca**^2+^** entry. The increase of [Ca**^2+^**]_i_ induced by KCa3.1 activation was drastically reduced by siKCa3.1 and siTRPV4. *n* = 10–20, ^∗^*p* < 0.05, ^∗∗^*p* < 0.01, one-way ANOVA followed by the Dunnett’s multiple comparison test compared with siCtrl. Ctrl, control.

To further study the molecular mechanism between TRPV4 and KCa3.1 in regulating asthmatic HBSM cells proliferation, we investigated the role of TRPV4 in KCa3.1-regulated Ca^2+^ influx. HBSM cells transfected with siTRPV4 or siKCa3.1 were used to confirm our hypothesis that there was functional coupling between TRPV4 and KCa3.1 channels. As mentioned above, the activation of KCa3.1 channels will hyperpolarize the membrane potential of HBSM cells and then enhance the calcium driving force. 1-EBIO, KCa3.1 specific agonist, induced [Ca^2+^]_i_ influx in the control HBSM cells which were abolished in Ca^2+^-free media (*p* < 0.05, **Figures 6A,B**). While in the HBSM cells transfected with siKCa3.1, the effect of 1-EBIO was reduced compared to control (*p* < 0.01, **Figures 6C,D**). We then investigated the role of TRPV4 in the process of Ca^2+^ influx induced by 1-EBIO-activated KCa3.1 channels. The HBSM cells transfected with siTRPV4 also showed a significant attenuation of Ca^2+^ influx induced by 1-EBIO (*p* < 0.01, **Figures 6C,D**). The basal Ca^2+^ concentrations of HBSM cells were decreased in both Ca^2+^-free media and transfected with siTRPV4 or siKCa3.1 (**Supplementary Figure S1**). It was also tested that mildly elevated K^+^ concentration (40 mM) counteracts the effects of 1-EBIO-induced Ca^2+^ influx (**Supplementary Figure S2**). Taken together, these data suggested that the HBSM cells membrane hyperpolarization-induced Ca^2+^ influx, due to activation of KCa3.1 channels, was via the TRPV4 channels.

### TRPV4 and KCa3.1 Co-localize in Asthmatic HBSM Cells

**FIGURE 7:**
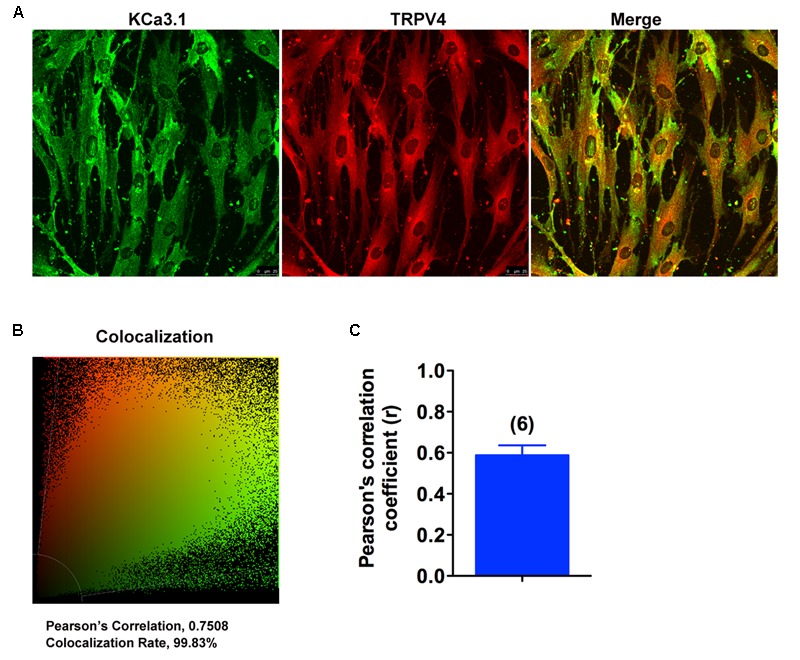
KCa3.1 and TRPV4 appear colocalized in asthmatic HBSM cells. **(A,B)** Double immunofluorescence images of KCa3.1 (green) and TRPV4 (red) in HBSM cells. Note the strong colocalization indicated by merge yellow fluorescence, quantification of the colocalization observed in experiments as shown in **(C)**. **(C)** The histograms represent the ratio of the mean Pearson correlation coefficient calculated from the colabeling in a number of samples, as indicated above the bar. Scale bar: 25 μm.

Colocalization experiments were conducted using specific KCa3.1 and TRPV4 antibodies. Confocal analysis of double-labeled staining showed that KCa3.1 and TRPV4 channels colocalized in HBSM cells (**Figures 7A,B**). As shown in **Figure 7C**, histograms represent the ratio of the mean Pearson correlation coefficient using Leica LAS AF Lite software. The Pearson correlation values range from -1 to +1. A correlation of 1 indicates complete colocalization between the two proteins. A correlation of -1 indicates a negative interaction, and a correlation of 0 indicates no colocalization between the two proteins. When double-labeled staining of KCa3.1 and TRPV4 channels, colocalization was observed in HBSM cells (**Figures 7A,B**). Pearson correlation coefficient is 0.59, confirming colocalization between KCa3.1 and TRPV4 (**Figure 7C**). Taken together, our results demonstrate that KCa3.1 and TRPV4 were colocalized in HBSM cells where they functional coupled and interacted in regulating Ca^2+^ entry.

### Both KCa3.1 and TRPV4 Are Involved in Asthmatic HBSM Cells Proliferation

Although proliferation of HBSM cells has been known to be a major characteristic feature of asthmatic airway remodeling, the mechanisms remain poorly understood. We have demonstrated that KCa3.1 was involve in cell cycle progression of asthmatic HBSM cells via membrane hyperpolarization and regulating Ca^2+^ influx (Yu et al., 2013b). The asthmatic HBSM cells proliferation can also be dependent on Ca^2+^ influx through TRPV4 channels (Dietrich et al., 2006). In this study, we investigated whether KCa3.1 and TRPV4 channels used a common pathway in regulating HBSM cells proliferation.

**FIGURE 8:**
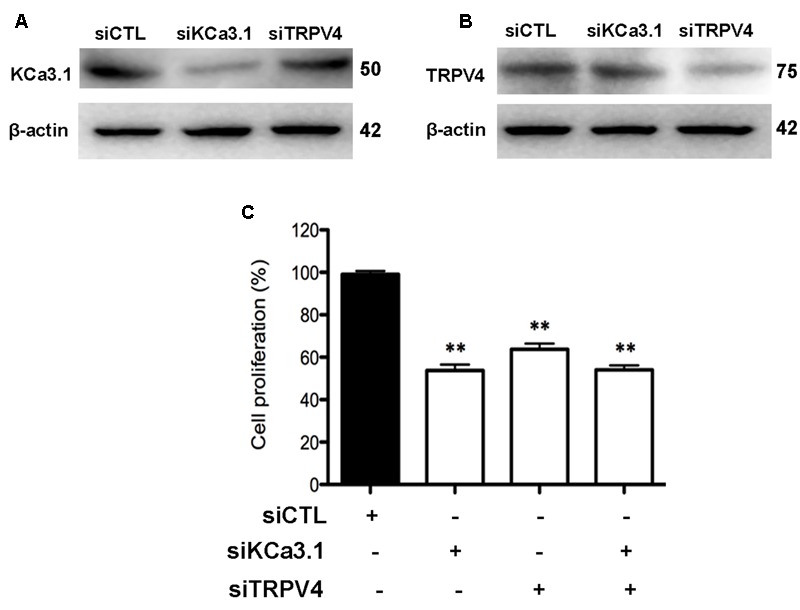
KCa3.1 and TRPV4 involvement in HBSM cells proliferation. **(A)** Representative western blot showing the effect of siRNAs directed against KCa3.1 and TRPV4 on the protein level of KCa3.1. **(B)** Representative western blot showing the effect of siRNAs directed against KCa3.1 and TRPV4 on the protein level of TRPV4. **(C)** Analysis of HBSM cells proliferation transfected with siCTL, siKCa3.1, or siTRPV4. Cell proliferation (CCK-8 assay) was measured as described in methods 72 h post-transfection. Values were reported as means ± SEM normalized to the control (*n* = 4). ^∗∗^*p* < 0.01, one-way ANOVA followed by the Dunnett’s multiple comparison test compared with siCTL. CTL, control.

HBSM cells were transfected with KCa3.1-specific siRNA (siKCa3.1) and TRPV4-specific siRNA (siTRPV4) and with scrambled siRNA as a negative control (siCTL). The efficacy of siKCa3.1 and siTRPV4 was firstly measured. The proteins expressions of TRPV4 and KCa3.1 were reduced following 72 h treatment with siKCa3.1 and siTRPV4, respectively (**Figures 8A,B**). Additionally, siTRPV4 did not affect the expression of KCa3.1 (**Figure 8A**) and neither did siKCa3.1 for the expression of TRPV4 (**Figure 8B**). We then measured the effect of KCa3.1 and TRPV4 silencing on the proliferation of HBSM cells using a CCK-8 assay. The proliferation of HBSM cells was significantly attenuated in the cells of transfected with either siTRPV4 (**Figure 8C**, *p* < 0.01) or siKCa3.1 (**Figure 8C**, *p* < 0.01) compared to siCTL. No synergistic effects were observed in HBSM cells transfected with both siTRPV4 and siKCa3.1 compared to the effects obtained with either siTRPV4 or siKCa3.1, respectively (**Figure 8C**, *p* < 0.01).

**FIGURE 9:**
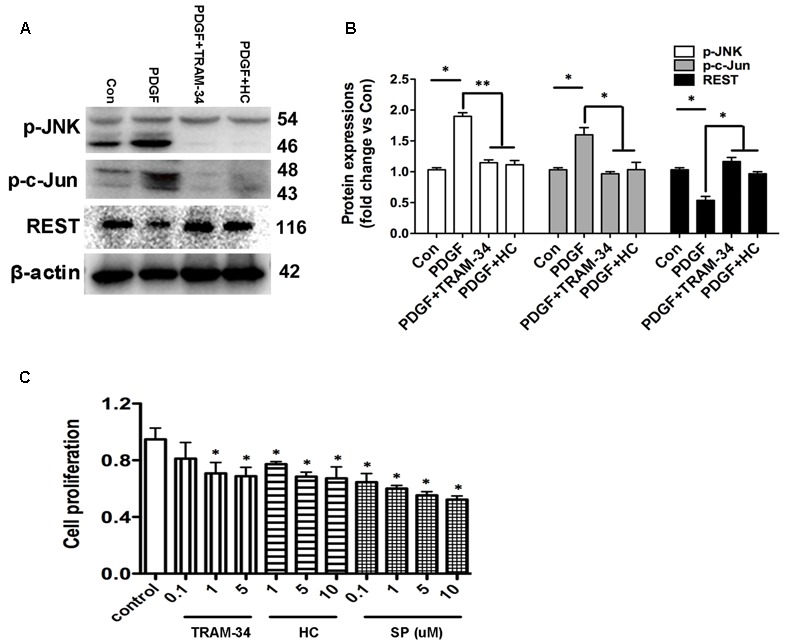
KCa3.1 and TRPV4 involvement in HBSM cells proliferation via JNK/c-Jun pathways. **(A)** p-JNK, p-c-Jun, and REST protein expressions in 1h PDGF stimulated HBSM cells with or without pretreatment of TRAM-34 (1 μM) or HC 067047 (10 μM). **(B)** Data were presented as means ± SEM. *n* = 5, ^∗^*p* < 0.05, ^∗∗^*p* < 0.01, one-way ANOVA followed by the Dunnett’s multiple comparison test compared with PDGF. Con, control; HC, HC 067047. **(C)** Analysis of HBSM cells proliferation treated with TRAM-34, HC 067047, or SP600125. Cells treated for 72 h with or without TRAM-34 (0.1–5 μM), HC 067047 (1–10 μM), or SP600125 (0.1–10 μM) in culture medium. Cell proliferation (CCK-8 assay) was then measured as described in methods. After treating cells with serum-free medium for 24 h, proliferation was induced by 10% FBS in the absence or presence of TRAM-34 (0.1–5 μM), HC 067047 (1–10 μM), or SP600125 (0.1–10 μM) for 72 h, and then assayed by measuring CCK-8 at the indicated times (*n* = 4). Values were reported as means ± SEM normalized to the control. ^∗^*p* < 0.05, one-way ANOVA followed by the Dunnett’s multiple comparison test compared with control. HC, HC 067047; SP, SP600125.

The expressions of JNK/c-Jun and REST proteins in the HBSM cells with or without the stimulation of PDGF were determined by Western blot analysis. As shown in **Figures 9A,B**, anti-JNK, anti-c-Jun and anti-REST antibodies were used to detect JNK, c-Jun and REST proteins from the 1h PDGF-stimulated HBSM cells with or without the pretreatment of TRAM-34 (1 μM) or HC 067047 (10 μM). Western blot analysis revealed that the expressions of JNK/c-Jun and REST were significantly higher and lower in PDGF stimulation than those in control group (**Figure 9B**, *p* < 0.05). In the pretreatment of TRAM-34 or HC 067047, blockade of KCa3.1 or TRPV4 decreased the expression of JNK/c-Jun and increased the expression of REST expression compared to the PDGF group (**Figures 9A,B**). We then measured the pharmacological blockade of KCa3.1 or TRPV4 channels, and inhibition of MAPK/JNK/c-Jun signal pathway on the effect of HBSM cells proliferation using a CCK-8 assay. The proliferation rate of HBSM cells was significantly attenuated in the cells pretreated with either TRAM-34 (0.1, 1, 5 μM) or HC 067047 (1, 5, 10 μM) compared to control group (**Figure 9C**). With the pretreatment of MAPK/JNK/c-Jun inhibitor SP600125 (0.1, 1, 5, 10 μM), the proliferation rate of HBSM cells was also significantly attenuated (**Figure 9C**, *p* < 0.05).

The simple scheme of positive feedback, whereby KCa3.1-induced hyperpolarization led to an increase in Ca^2+^ influx via TRPV4, and the gene expressions of REST and AP-1 were correlated negatively or positively with the expression of KCa3.1 channels (**Supplementary Figure S3**).

## Discussion

The major findings in this study were 1. Gene deletion of KCa3.1 resulted in an attenuation of AHR, airway inflammation, and airway remodeling in a mouse model of chronic asthma, and 2. KCa3.1 regulated Ca^2+^ influx and cells proliferation by functional cooperation with TRPV4 in asthmatic HBSM cells. Silencing either KCa3.1 or TRPV4 attenuated proliferation of asthmatic HBSM cells, and reduced Ca^2+^ influx via KCa3.1 activation. Additionally, colocalization experiments showed that TRPV4 and KCa3.1 were colocalized in the HBSM cells. These data suggested that KCa3.1 channels might represent a potential target for human allergic asthma.

As a key feature of chronic asthma, airway remodeling is characterized by airway SMC hyperplasia and matrix deposition in the airways (Grzela et al., 2016; Zhang et al., 2016). As a major contributor to airway remodeling, the mechanisms of airway SMC proliferation remain unknown. Proliferation of SMC requires a phenotype switch from the normally contractile state to a synthetic phenotype during chronic asthma (Johnson et al., 2001; Khan, 2013). The mechanisms underlying the phenotype switch process are not fully understood.

In non-excitable cells such as SMC, the lack of voltage-gated Ca^2+^ channels allows KCa3.1 channels to act as both Ca^2+^ detectors and Ca^2+^ amplifiers, whereby activation of KCa3.1 induced K^+^ efflux and membrane hyperpolarization ultimately up-regulated the driving force for Ca^2+^ influx (Gueguinou et al., 2014, 2015). TGFβ1-stimulated the activation of KCa3.1 contributed to α-SMA increase via phosphorylation of Smad, and also contributed to kidney fibrosis in rodents (Huang et al., 2013). KCa3.1 was involved in proliferation, chemotaxis, and activation of some immune and non-excitable cells including airway SMC, via regulating a negative membrane potential, thus increasing the driving force for Ca^2+^ influx (Henriquez et al., 2016; Chiang et al., 2017).

There are now many evidences that KCa3.1-Ca^2+^ channel complexes are found in non-excitable cells and contribute to cells-associated proliferation and migration functions (Gueguinou et al., 2014). TRP channels have been obvious candidates for the Ca^2+^ influx in airway SMC. Both TRPV2 and TRPV4 channels were reported to present in primary HBSM cells (Jia et al., 2004). Although Jia et al. (2004) showed strong evidences for TRPV4 channel in hypotonicity-induced bronchial contraction via the extracellular Ca^2+^, the role of TRPV2 channels couldn’t be completely excluded. Yu et al. (2004) also showed that HBSM cells expressed TRPC1, -4, and -6 channels. It might explain the reason that silencing of KCa3.1 has a stronger effect on the response to 1-EBIO than silencing of TRPV4 as shown in **Figure 6**. An interaction between KCa3.1 and TRPV4 channels has been found in several diseases, during which Ca^2+^ dynamics induced by KCa3.1 were dependent on Ca^2+^ influx via TRPV4 channels (Qian et al., 2014; Li et al., 2017). KCa3.1 channels may amplify mesenteric arteries vasodilation by expanding TRPV4-induced Ca^2+^ influx. Wandall-Frostholm et al. (2015) recently reported that KCa3.1 deficiency attenuated pulmonary circulatory collapse via activation of TRPV4 channel (2015). There was a functional coupling between KCa3.1 and TRPV4 to regulate Ca^2+^ levels leading to pulmonary circulatory collapse and hemorrhage (Simonsen et al., 2017). KCa3.1 regulated proliferation of human prostate cancer cell via Ca^2+^ influx through TRPV6 (Lallet-Daher et al., 2009). There was an endogenous interaction between KCa3.1 and TRPC1, which allowed TRPC1 controlling the Ca^2+^ influx via KCa3.1 activation, promoting human breast cancer cells proliferation (Faouzi et al., 2016).

KCa3.1 involves in proliferation of immune cells, tumors, and undifferentiated smooth muscles and represents a potential target for autoimmune disease, cancer, and restenosis (Wulff et al., 2007). The gene expressions of REST and AP-1 were correlated negatively or positively with the expression of KCa3.1 channels both *in vivo* and *in vitro* (Ghanshani et al., 2000; Cheong et al., 2005; Tharp et al., 2008). In response to differential stimuli, AP-1 is involved in cellular processes including proliferation, differentiation, and apoptosis. In this study, c-Jun has been shown significant up-regulation in both the gene and protein levels but not others (c-Fos, FosB, Fra1, Fra2, JunB, and JunD) in WT + OVA group compared to the WT group. Previously, we reported that blockade or gene deficiency of KCa3.1 attenuated HBSM cell migration and proliferation via decrease in [Ca^2+^]_i_. It was shown that pharmacological blockade of KCa3.1 prevented mitogen-induced enrichment of the KCa3.1 promoter with c-jun. The mechanism for KCa3.1 up-regulation is likely dependent on AP-1. It has been reported that delivery of REST to proliferating cells could repress KCa3.1 expression, illustrating the downstream mechanism of REST regulates KCa3.1 expression. It is also hypothesized that the KCa3.1 activity-induced membrane hyperpolarization or Ca^2+^ influx drives down-regulation of REST expression (Cheong et al., 2005).

These results demonstrate that KCa3.1 is involved in the phenotype switch of HBSM cells via TRPV4 channels in the process of chronic asthma, making it a potential therapeutic target to treat chronic asthma.

## Author Contributions

ZY supervised the entire project, designed research, and wrote the paper. HC supervised all the experimental procedure. YW and LQ performed researches and analyzed data.

## Conflict of Interest Statement

The authors declare that the research was conducted in the absence of any commercial or financial relationships that could be construed as a potential conflict of interest.

## Supplementary Material

FIGURE S1 TRPV4 involved in Ca^2+^ entry induced by KCa3.1 activation in asthmatic HBSM cells. **(A,B)** Representative curves showed the fluorescence intensity in [Ca^2+^]_i_ over 360 s. **(A)** External calcium was required for 200 μM 1-EBIO-induced Ca^2+^ elevations. The increase of [Ca^2+^]_i_ induced by 200 μM 1-EBIO was prevented by 0 external Ca^2+^. **(B)** Representative traces of 1-EBIO-induced Ca^2+^ entry in the HBSM cells transfected with siKCa3.1, siTRPV4 or siCtrl. 1-EBIO was used at 200 μM. Ctrl: control.

FIGURE S2 1-EBIO induced Ca^2+^ entry with or without external K^+^ in asthmatic HBSM cells. **(A,B)** Representative curves showed the fluorescence intensity in [Ca^2+^]_i_ over 360 s. **(A)** 200 μM 1-EBIO-induced Ca^2+^ elevations. **(B)** The increase of [Ca^2+^]_i_ induced by 200 μM 1-EBIO was prevented by pretreatment of 40 mM external K^+^.

FIGURE S3 The gene expressions of REST and AP-1 were correlated negatively or positively with the expression of KCa3.1 channels. The gene expressions of REST and AP-1 were correlated negatively or positively with the expression of KCa3.1 channels in chronic asthma. KCa3.1 regulated Ca^2+^ influx via functional cooperation with TRPV4 in asthmatic HBSM cells.

## Abbreviations

1-EBIO: 1-ethylbenzimidazolinone
AHR: airway hyperresponsiveness
AP-1: activation protein-1
DAPI: 2-(4-Amidinophenyl)-6-indolecarbamidine dihydrochloride
HBSM: human bronchial smooth muscle
JNK: c-Jun N-terminal kinase
KCa3.1: intermediate-conductance calcium-activated potassium channel
PDGF: platelet-derived growth factor
REST: repressor element 1-silencing transcription factor
SMC: smooth muscle cells
TRAM-34: 1-((2-chlorophenyl) (diphenyl) methyl)-1H-pyrazole
TRPV4: transient receptor potential vanilloid 4
