# Supplementary figures and images for: Functional Cooperation between KCa3.1 and TRPV4 Channels in Bronchial Smooth Muscle Cell Proliferation Associated with Chronic Asthma
Source: Front Pharmacol. 2017 Aug 25;8:559. doi: 10.3389/fphar.2017.00559 (PMC5609593; doi:10.3389/fphar.2017.00559)

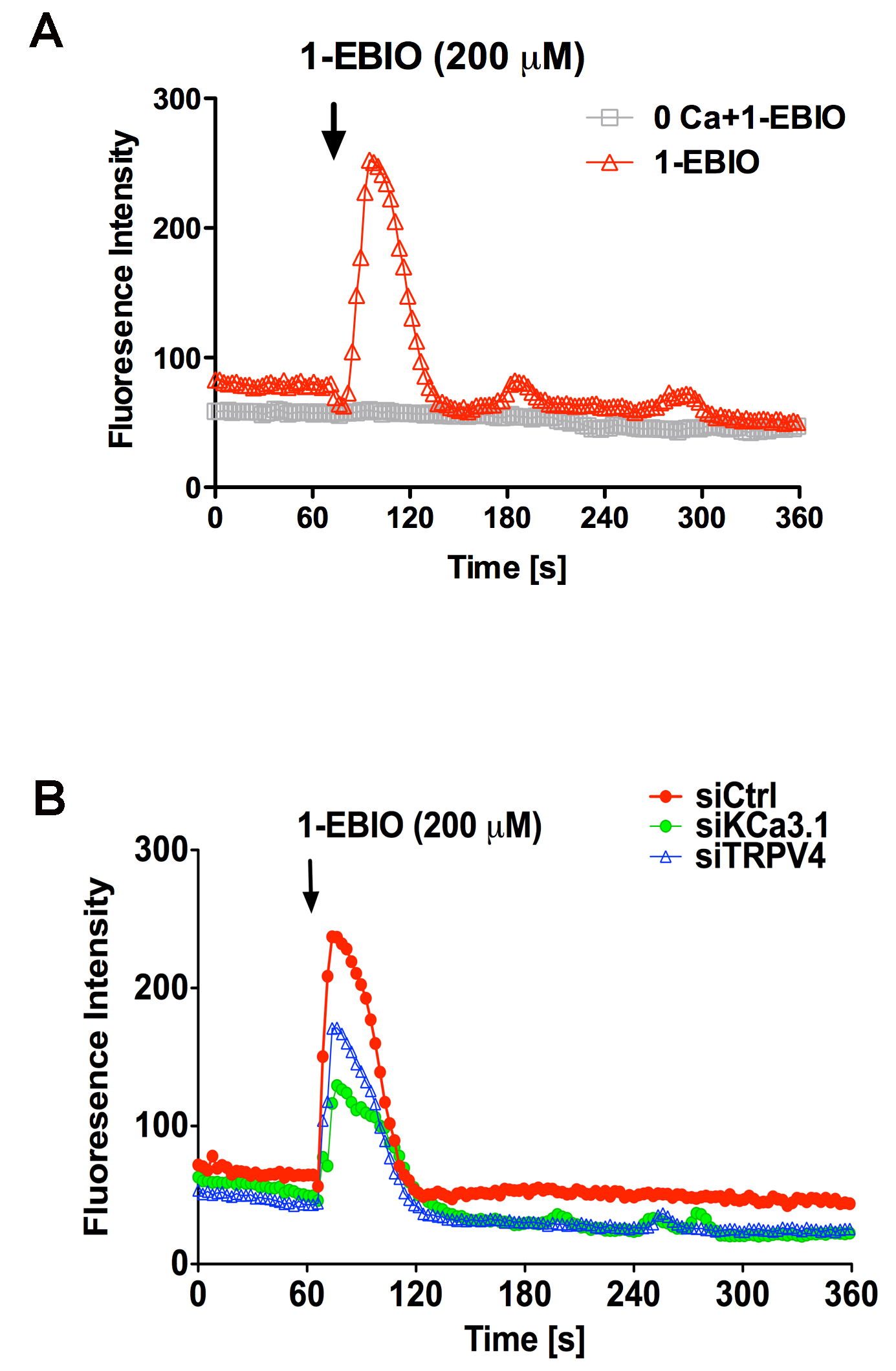

Supplement: FIGURE S1 — TRPV4 involved in Ca2+ entry induced by KCa3.1 activation in asthmatic HBSM cells. (A,B) Representative curves showed the fluorescence intensity in [Ca2+]i over 360 s. (A) External calcium was required for 200 μM 1-EBIO-induced Ca2+ elevations. The increase of [Ca2+]i induced by 200 μM 1-EBIO was prevented by 0 external Ca2+. (B) Representative traces of 1-EBIO-induced Ca2+ entry in the HBSM cells transfected with siKCa3.1, siTRPV4 or siCtrl. 1-EBIO was used at 200 μM. Ctrl: control. [file Image_1.TIF]

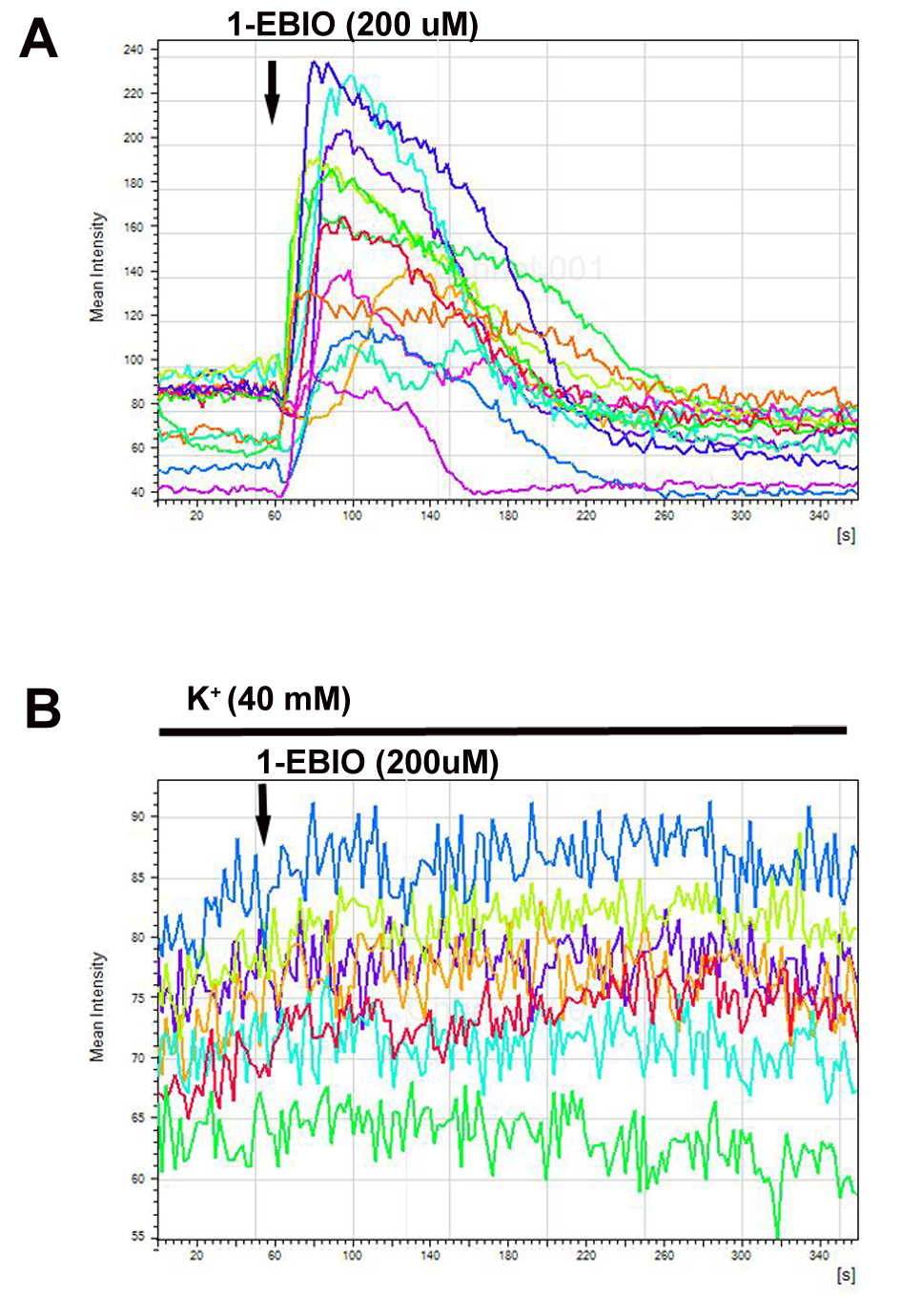

Supplement: FIGURE S2 — 1-EBIO induced Ca2+ entry with or without external K+ in asthmatic HBSM cells. (A,B) Representative curves showed the fluorescence intensity in [Ca2+]i over 360 s. (A) 200 μM 1-EBIO-induced Ca2+ elevations. (B) The increase of [Ca2+]i induced by 200 μM 1-EBIO was prevented by pretreatment of 40 mM external K+. [file Image_2.TIF]

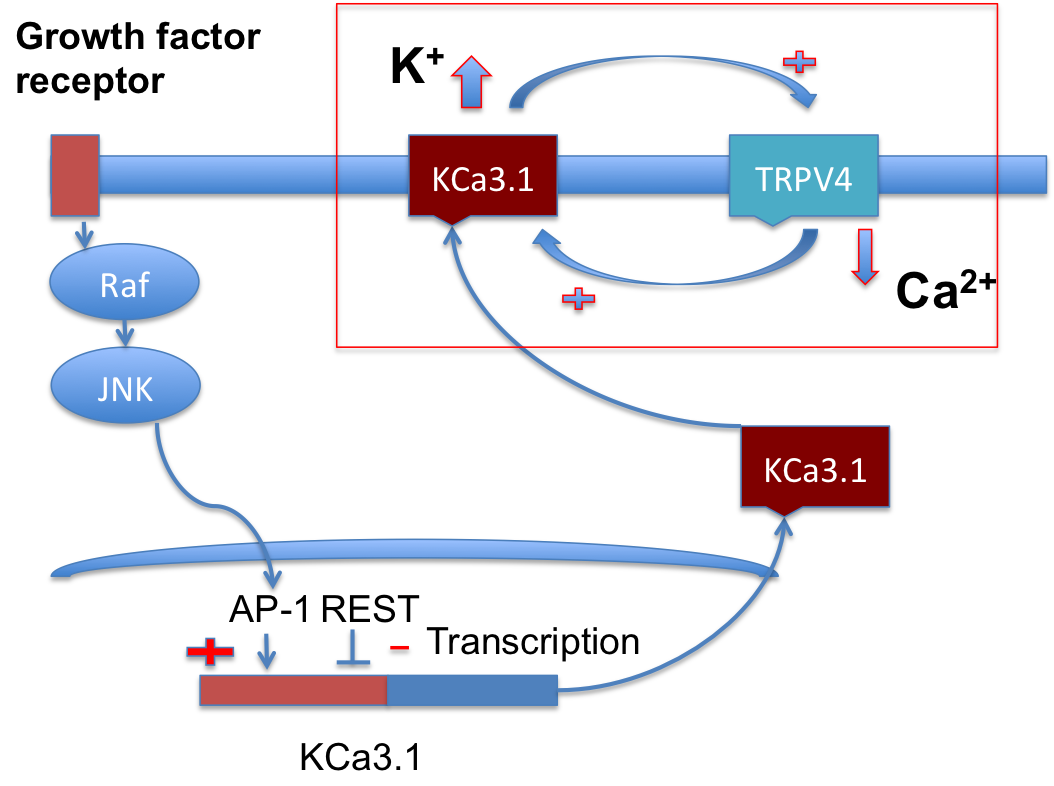

Supplement: FIGURE S3 — The gene expressions of REST and AP-1 were correlated negatively or positively with the expression of KCa3.1 channels. The gene expressions of REST and AP-1 were correlated negatively or positively with the expression of KCa3.1 channels in chronic asthma. KCa3.1 regulated Ca2+ influx via functional cooperation with TRPV4 in asthmatic HBSM cells. [file Image_3.TIF]
